# Therapeutic Efficacy of Small Extracellular Vesicles Loaded with ROCK Inhibitor in Parkinson’s Disease

**DOI:** 10.3390/pharmaceutics17030365

**Published:** 2025-03-13

**Authors:** Candy Carbajal, Myosotys Rodriguez, Florida Owens, Nicole Stone, Dileepkumar Veeragoni, Rebecca Z. Fan, Kim Tieu, Nazira El-Hage

**Affiliations:** 1 Department of Cellular and Molecular Medicine, Herbert Wertheim College of Medicine, Florida International University, Miami, FL 33199, USA; ccarb060@fiu.edu (C.C.); myrodrig@fiu.edu (M.R.); fowen008@fiu.edu (F.O.); nston006@med.fiu.edu (N.S.); dveerago@fiu.edu (D.V.); 2 Department of Environmental Health Sciences, Robert Stempel College of Public Health & Social Work, Florida International University, Miami, FL 33199, USA; zhfan@fiu.edu (R.Z.F.); ktieu@fiu.edu (K.T.)

**Keywords:** drug delivery system, intranasal delivery, extracellular vesicles, Parkinson’s disease, ROCK

## Abstract

**Background/Objectives:** Parkinson’s disease (PD) is a rapidly growing neurological disorder in the developed world, affecting millions over the age of 60. The decline in motor functions occurs due to a progressive loss of midbrain dopaminergic neurons, resulting in lowered dopamine levels and impaired muscle function. Studies show defective mitochondrial autophagy (or “mitophagy”) links to PD. Rho-associated coiled-coil containing protein kinases (ROCK) 1 and ROCK2 are serine/threonine kinases, and their inhibition can enhance neuroprotection in PD by promoting mitophagy. **Methods:** We examine the effects of ROCK inhibitor SR3677, delivered via macrophage-derived small extracellular vesicles (sEVs) to Parkin Q311X(A) PD mouse models. sEVs with SR3677, administered intranasally, increased mitophagy gene expression, reduced inflammatory factors, and elevated dopamine levels in brain tissues. **Results:** ROCK2 expression decreased, showing the drug’s inhibitory effect. sEV-SR3677 treatment was more effective than treatment with the drug alone, although sham EVs showed lower effects. This suggests that EV-SR3677 not only activates mitochondrial processes but also promotes the degradation of damaged mitochondria through autophagy. Mitochondrial functional assays and oxygen consumption in ex vivo glial cultures revealed that sEV-SR3677 significantly improved mitochondrial respiration compared to that in untreated or SR3677-only treated cells. **Conclusion:** We demonstrated the efficacy of ROCK2 inhibition on mitochondrial function via sEV-SR3677 in the PD mouse model, necessitating further studies to explore design challenges and mechanisms of sEV-SR3677 as mitochondria-targeted therapy for PD

## 1. Introduction

Parkinson’s disease (PD) is recognized as a progressive neurodegenerative disorder, characterized by the inevitable decline of dopaminergic neurons in the substantia nigra [1], along with abnormal intracellular aggregation of proteins such as α-synuclein [2]. The loss of neurons is believed to result from improper management of misfolded proteins, mitochondrial dysfunction, disruption of the autophagic–lysosomal system, endoplasmic reticulum stress [3], and imbalanced calcium levels [1]. Abnormal α-synuclein accumulation may accelerate disease progression by inducing excessive or dysregulated ER stress responses. In reaction, the unfolded protein response activates the expression of Parkin as a protective measure. By preventing the buildup of Parkin substrates, the elevated levels of Parkin that result from this process help shield neurons from cell death triggered by ER stress [3]. Additionally, the activation of microglia, the resident immune cells in the central nervous system (CNS), and the release of various proinflammatory cytokines—particularly TNF-α, IL-1β, and IL-6—have been observed in the brains of individuals affected by PD. The accumulation of damaged mitochondria within the substantia nigra pars compacta (SNpc) dopaminergic system is another hallmark of PD. Defective mitochondrial autophagy, or “mitophagy”, along with an accumulation of autophagosomes, has been documented in PD brains, indicating a decline in successful clearance in this disease [4]. Thus, mitochondria-targeted drug therapies represent innovative strategies to enhance a crucial disease mechanism in PD.

Rho-associated coiled-coil containing protein kinases (ROCK) 1 and ROCK2 are universal isoforms belonging to the serine/threonine kinase family, which play roles in actomyosin cytoskeleton organization, cell migration, apoptosis, and the contraction of smooth muscle cells [5,6,7,8]. ROCK1 is found throughout the bodies of mice, including in the brain, but is more prominently expressed in non-neuronal tissues [9,10]. Conversely, ROCK2 mRNA is highly expressed in the brain, muscle, heart, lung, and placenta, suggesting distinct functions of each isoform in these tissues [11]. Research suggests that ROCK2 may be more prominently involved in PD due to its higher expression in specific brain regions affected by the disease [12]. Published research has contributed to a growing body of evidence that ROCKs are crucial therapeutic targets for cardiovascular disease [8], diabetic kidney disease [13], and neurological disorders, with ROCK inhibitors proposed as novel therapeutics in various clinical scenarios [14].

SR3677, a selective drug inhibitor of ROCK2, was investigated for its role in modulating mitophagy in a Parkinson’s disease model. It activated Hexokinase-2, a positive regulator of Parkin, which enhanced its recruitment to damaged mitochondria. This, in turn, triggered PINK1–Parkin-mediated mitophagy and provided neuroprotective effects in Drosophila exposed to the Parkinsonian toxin paraquat [15]. Downregulation of ROCK2 was found to protect dopaminergic neurons in the substantia nigra from 6-OHDA-induced degeneration and resulted in a significant increase in the number of Tyrosine Hydroxylase (TH)-positive neurons [16], a dopaminergic neuron marker [17]. However, delivering these small-molecule therapeutics to the brain presents considerable challenges due to the blood–brain barrier, a complex multicellular structure composed of endothelial cells, pericytes, astrocytes, and microglia [18,19].

We have previously demonstrated the successful use of macrophage-derived extracellular vesicles (EVs) as biologically active nanocarriers in PD [20,21]. Circulating EVs may function as inflammatory mediators and carriers of oxidative stress signals. They have been used to demonstrate that EVs and their adipose renin–angiotensin system (RAS) cargo serve as a mechanistic link between metabolic syndrome (MetS) and PD. Notably, MetS promotes low-grade peripheral oxidative stress, inflammation, and RAS dysregulation, all of which are implicated in the progression of dopaminergic degeneration and PD [22]. EVs serve as both short- and long-distance mediators of intercellular communication, offering distinct advantages that uniquely position them as highly effective drug carriers. These advantages include high stability, bioavailability, low immunogenicity, biocompatibility, and significant biological activity [23,24]. These small, membrane-bound vesicles are secreted by cells and contain various biomolecules, including nucleic acids, proteins, and lipids. Due to their biological origin, EVs are safer, less immunogenic, and possess specific surface proteins that help them avoid entrapment in the mononuclear phagocyte system, thus facilitating the delivery of their therapeutic cargo to the targeted site. EVs are small enough to cross the blood–brain barrier (BBB) [25,26], making them ideal for targeting specific brain regions. Furthermore, they naturally target and enter specific cells, enhancing drug delivery efficiency and reducing the required dosage through less invasive administration routes such as intravenous, intranasal, and intraperitoneal methods [27,28,29]. Recently, EVs have been shown to exhibit even greater advantages as drug delivery vehicles due to their exceptional ability to cross biological barriers and avoid accumulation in peripheral organs [30]. Here, we utilized macrophage-derived sEVs loaded with SR3677, which was administered intranasally to the brain of Parkin Q311X(A) mice. Our pilot study demonstrated how inhibiting ROCK2 enhances mitochondrial function and increases dopamine levels via sEV-SR3677 in a PD mice model.

## 2. Materials and Methods

### 2.1. Animal Model and Treatments with EV-SR3677

Small extracellular vesicles (sEVs) derived from macrophages were extracted from conditioned media through a sequential centrifugation process. This involved two spins at 167,000× *g* for four hours to collect the supernatant, followed by another spin at 167,000× *g* for 19 h. The resulting pellet was re-suspended in saline buffer and characterized based on the guidelines outlined in MISEV2018, focusing on size, morphology, and protein content, as described in Refs. [31,32]. SR3677 was purchased from Selleckchem (Houston, TX, USA). To incorporate SR3677 into the EVs, drug stock solution (10 mg/mL) in DMSO was added to an sEV suspension on ice and sonicated under the following conditions: 20% amplitude, with 4 s on/8 s off × 12 cycles. Parkin Q311X(A) male mice (16 months old, N = 5) were treated with sEV-SR3677 (5 × 10^10^ particles/20 μL/mouse), corresponding to the following doses of the SR3677 (3.75 mg/kg), via intranasal administrations three times every other day, as previously described [31,33]. Additional control groups of PD male mice (N = 5) received either saline, sham sEVs, or the drug alone (at the same doses). At the endpoint (4 h after the third administration), the animals were deeply anesthetized with sodium pentobarbital (12 mg/kg), euthanized, and perfused, and the brains, kidneys, livers, and spleens were recovered and stored, as previously described [34]. For primary murine glial culture, C57BL/6J littermates were sacrificed and cultured from postnatal days 4 to 6 (P4-P6), as previously described [35]. The mice were purchased from the Jackson Laboratory (Bar Harbor, ME, USA) and cared for according to the Principles of Animal Care outlined by the National Institutes of Health, approved by the Institutional Animal Care and Use Committee of Florida International University, protocol approval number (IACUC #18-030 and #22-067).

### 2.2. Reverse Transcription Polymerase Chain Reaction (RT-PCR)

Total RNA was extracted from mouse postmortem brain tissues using TRIzol Reagent (Life Technologies, Carlsbad, CA, USA) and was used to measure the expression of 84 transcription factor genes using the RT2 Profiler PCR Arrays (Qiagen, Hilden, Germany), as described previously by us [36]. The CT data were interpreted according to a manufacturer’s website. Relative expression was calculated according to the ΔΔCT method, using five housekeeping genes, and compared with that in the non-treated control brains.

### 2.3. Western Blotting

SDS-PAGE was used to separate the proteins (25 µg) in the postmortem brain homogenates, which were then transferred to the PVDF membranes, and they were subsequently incubated with the following antibodies: Beclin1 (catalog # ab62557), PARKIN (catalog # ab77924), PINK (catalog # ab23707), which were procured from Abcam(Waltham, MA, USA), and used at 1:1000 dilution. TOM 20 (catalog # MA5-32148), obtained from Invitrogen (Carlsbad, CA, USA); Caspase 3 (catalog # 9665), from Cell Signaling Technology (Danvers, MA, USA); P62/SQSTM1 (catalog # NBP1-42821SS), procured from Novus Biologicals (Centennial, CO, USA), were used at a 1:1000 dilution, and β-actin (internal control catalog #sc47778), obtained from Santa Cruz Biotechnology (Dallas, TX, USA), was employed at a 1:200 dilution. Membranes were incubated with horseradish peroxidase-conjugated secondary antibodies, specifically the anti-mouse IgG HRP-linked antibody (catalog # 7076) from Cell Signaling Technology and the anti-Rabbit IgG (catalog # A0545) from Sigma-Aldrich (Saint Louis, MO, USA), both used at a 1:1000 dilution. This was followed by exposure to SuperSignal West Femto Substrate (Thermo Scientific, Waltham, MA, USA) and visualization with a ChemiDoc imaging system from Bio-Rad, with further quantification using ImageJ software version 1.52 from NIH.

### 2.4. Enzyme-Linked Immunosorbent Assay (ELISA)

Cytokines, including interleukin 6 (IL-6), chemokines monocyte chemotactic protein-1 (MCP-1), and regulated on activation, normal T cell expressed and secreted (RANTES), were measured in postmortem brain, liver, kidney, and spleen homogenates using an individual sandwich ELISA kit from R&D Systems (Minneapolis, MN, USA), as per the manufacturer’s protocol. The optical density was read at A450, with wavelength correction at A570 on a Synergy HTX plate reader from BioTek (Winooski, VT, USA).

### 2.5. Hematoxylin and Eosin (H&E) Staining

Tissue slides were sectioned to a thickness of 12 microns and briefly rewarmed at room temperature for 30 min to remove the embedding compound, then stained with hematoxylin dye for 15 min, followed by a wash with distilled water. The tissue slides were stained with eosin for 20 s, dehydrated using a gradient of ethanol (70%, 95%, 100%) after washing with tap water, cleared with xylene, and mounted with mounting media for visualization. Images were analyzed using an inverted fluorescence microscope from Zeiss(Germany), equipped with a 560 Axiovision camera at 40×.

### 2.6. Matrix-Assisted Laser Desorption/Ionization Time-of-Flight (MALDI-TOF) Mass Spectrometry (MS)

Certified standards of dopamine and dopamine-D4 were obtained from Cerilliant (Round Rock, TX, USA) (1000 mg/L) and stored in the dark at −20 °C until use. These standards were serially diluted in acetonitrile and utilized without further treatment. Amber glass LC vials with polypropylene and spring-loaded inserts were capped with PTFE-coated septa. Control samples for each tissue were employed to assess method performance. Tissue samples were stored in the dark at −80 °C until use. Approximately 50 mg of tissue was weighed in a centrifuge tube, and then 40 μL of 50 ng/mL dopamine-D4 was added. The volume was adjusted to 2 mL with the extraction solution (ethyl acetate and isopropanol *v/v* 6:4). The sample was swirled and mixed for 10 s and then underwent ultrasonic extraction. After centrifugation at 2500× *g* for 10 min at 4 °C, the supernatant was transferred to a new tube and dried under nitrogen. The sample was then redissolved in 200 μL methanol and analyzed using HPLC-MS. For the LC step, a sample injection of 40 μL was administered, and LC separation was performed using a Shimadzu Prominence LC-20 CE Ultra-Fast Liquid Chromatograph (Shimadzu, Japan). A Waters Xbridge BEH Phenyl Column (4.6 mm × 150 mm, 3.5 μm), protected by a VanGuard cartridge (3.9 mm × 5 mm, 3.5 μm), was the analytical column used. A mobile phase gradient was created using Optima LCMS-grade water and Optima LCMS acetonitrile, both containing 0.1% formic acid. The total running time was 15 min. MS/MS detection was performed via LC-TIMS-TOF MS/MS analysis conducted on a Bruker (Billerica, MA, USA) equipped with a heated VIP-HESI ionization source, operating under positive electrospray. The calibration curve (Supplementary Material Figure S1) included six concentrations in ng/mL (ppb), i.e., CS1: 0.1 ng/mL; CS2: 0.5 ng/mL; CS3: 5 ng/mL; CS4: 20 ng/mL; CS5: 100 ng/mL; CS6: 500 ng/mL.

### 2.7. Mitochondria Bioenergetics

The extracellular acidification rate (ECAR) and the oxygen consumption rate (OCR) were measured using a Seahorse XF96 Extracellular Flux Analyzer (Seahorse Biosciences Inc, Agilent, Santa Clara, CA, USA) [37]. Primary murine glia cells (1 × 10^6^ cells per well) were seeded in an XF96 plate, and the number of cells per well was 75,000/0.32 cm^2^. The cells were exposed to SR3677 or EV-SR3677 (5 μM for 4 h). The XF96 culture microplates were incubated in a CO_2_-free XF incubator at 37 °C for 45 min for temperature and pH calibration. For the Mito Stress test, oligomycin (1 μg/mL final concentration), carbonyl cyanide 4-(trifluoromethoxy) phenylhydrazone (FCCP, 0.5 μM final concentration), and rotenone plus antimycin A (0.5 μM final concentration of each) were sequentially injected. These agents were used to assess basal mitochondrial respiration, reserve respiratory capacity, and maximal respiratory capacity measurements in pmoles/min of oxygen consumed. Seahorse Wave software version 2.2.0 (Agilent)analyzed the data. ECAR was recorded in mpH/min [38].

### 2.8. Statistical Analysis

The results are reported as mean ± SEM from three to five independent experiments. Data were analyzed using analysis of variance (ANOVA), followed by post hoc Tukey’s test for multiple comparisons with GraphPad Prism 7.02 (GraphPad Software, Boston, MA, USA). A *p*-value of <0.05 was deemed significant.

## 3. Results

### 3.1. sEVs-SR3677 Upregulated the Transcription of Genes and Proteins Responsible for Mitochondrial Function and the Mitophagy Pathway in the PD Mouse Brain

**Figure 1:**
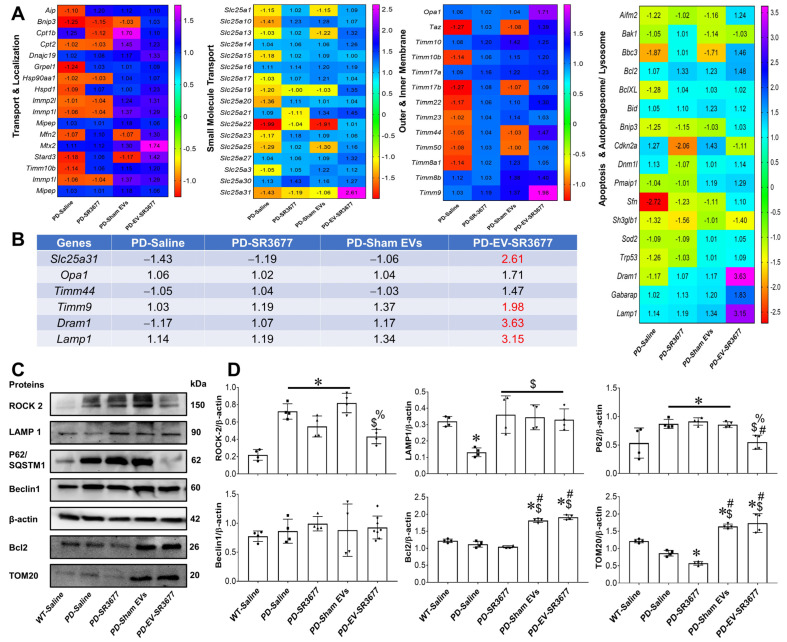
Upregulation of genes and proteins associated with mitophagy and mitochondrial function. RNA extracted from necropsied brains recovered from healthy C57BL6/J male mice treated with saline and Parkin Q311X male mice treated with saline, SR3677, sham EVs, and EV-SR367 was used to measure the expression of 84 mitophagy-related genes by RT-PCR (**A**). Relative expression was calculated using the ΔΔCT method, with five housekeeping genes, and compared with those in non-treated control brains. The most noticeable genes that increased by 1.5-fold or higher are indicated in red in the small table (**B**). Proteins extracted from necropsied brains were used via Western blotting to measure the expression of ROCK 2, LAMP1, P62/SQSTM1, Beclin1, Bcl2, TOM20, with β-actin as an internal control (**C**,**D**). Membranes were visualized using a ChemiDoc imaging system from Bio-Rad and quantified using Image J software from NIH. Results are reported as the mean ± SEM of three independent experiments. Data were analyzed using one- or two-way ANOVA analysis, followed by Tukey’s multiple comparisons test. A value of *p* < 0.05 was considered significant. * vs. WT-Saline; ^$^ vs. PD-Saline; ^#^ vs. PD-SR3677; ^%^ vs. PD-sham EVs.

The therapeutic efficacy of sEV-SR3677 was evaluated in 16-month-old Parkin Q311X(A) mice, which correlate to 54-year-old human adults [39]. For drug loading, sEVs were treated with SR3677 using sonication (as described in the Methods section), and then purified from the unincorporated drug via size-exclusion chromatography. Nanoparticle tracking analysis (NTA) data indicated that the average size of sEV-SR3677 was 30.1 ± 5.4 nm, with a concentration of 7.8 × 10^12^ particles/mL, achieving approximately 94% recovery. The zeta potential (Z) was minimally affected by drug loading, measuring −21.0 ± 0.7 mV (Supplementary Material Table S1). The sonication method’s loading efficiency (LE%) was ~45%. We hypothesized that a relatively high LE% was achieved due to the large ratio of surface vs. internal cavity in sEVs, allowing a significant amount of the hydrophobic drug SR3677 to be incorporated into a lipid membrane. Recovered brains obtained at necropsy were divided into two hemispheres. Half were homogenized, and RNA extracts were used to measure the expression levels of key mitophagy-related genes by RT-PCR. Of the 84 genes expressed, PD control mice treated with saline showed downregulation of various genes involved in mitochondrial function and mitophagy (Figure 1A,B). For instance, *Aip*, *Bnip3*, and *Grpel1*—genes involved in the localization and transport of proteins to the mitochondria—were significantly downregulated in PD animals treated with saline compared to the results for wild-type controls. In contrast, the gene expressions of *Mxt2* and *Stard3* in PD animals treated with sEV-SR3677 were upregulated by 1.7-fold and 1.5-fold, respectively. Interestingly, animals treated with sham sEV showed upregulation of *Cpt1b* by 1.7-fold and *Cpt2* by 1.5-fold, and these are involved in mitochondrial lipid metabolism. Gene expression analysis indicated that *Slc25a22*, which encodes a mitochondrial glutamate transporter protein, was downregulated 2-fold in PD animals treated with saline and sham sEVs. Conversely, *Slc25a31*, which encodes a protein that exchanges cytosolic ADP for matrix ATP in the mitochondria, was upregulated by 2.6-fold in animals treated with sEV-SR3677. Cells overexpressing this gene have been shown to display an anti-apoptotic phenotype [2,4]. Compared to the results for wild-type (control) animals, various genes involved in outer and inner membrane translocation, such as *Taz*, *Timm17b*, *Timm44*, and *Timm50*, were downregulated in PD animals treated with saline and sham sEVs. However, compared to saline-treated control PD brains, in animals treated with sEV-SR3677, the gene *Opa1*, which encodes the optic atrophy protein 1 (OPA1) that plays a crucial role in mitochondrial structure, fusion, and stability, and *Timm9,* which encodes a protein that inserts proteins into the mitochondrial inner membrane, were upregulated by 1.7-fold and 2-fold, respectively. Gene expression of apoptosis and autophagosome/lysosome related genes showed that compared to the results for the wild type animals, the expression of the *Sfn* gene, which encodes the protein stratifin, was downregulated in PD brains treated with saline, PD SR3677, and sham sEVs by 3-fold and 1-fold, respectively. Conversely, compared to PD saline treated animals, in brains treated with sEV-SR3677, the expression of the *Sfn* gene was upregulated. Also, the gene *Bbc3*, which is an important regulator of apoptosis and plays a role in various diseases, including Parkinson’s disease, was downregulated by 2-fold in PD brains treated with saline and sham sEVs and upregulated in the brains of PD animals treated with SR3677 alone and sEV-SR3677. Moreover, the cyclin-dependent kinase inhibitor 2A, *Cdkn2a*, was downregulated in the brains of PD animals treated with SR3677 alone and sEV-SR3677 by 2-fold and 1-fold respectively. In contrast, compared to the results for PD animals treated with saline, the gene expression of *Dram*1, *Gabarap*, and *Lamp1* was upregulated in brains of PD animals treated with sEV-SR3677 by 4-, 2-, and 3-fold, respectively.

Overall, these findings indicated that the effect of sEV-SR3677 treatment on the upregulation of mitochondrial function and mitophagy-related genes in brain tissues was greater than that observed for the drug alone. Sham EV also produced some effects but at considerably lower levels. This suggests that, along with activating mitochondrial processes, sEV-SR3677 can stimulate the degradation of damaged or accumulated mitochondria via autophagy (or mitophagy) mechanisms (Figure 1A). This is indeed of interest, as the dysregulation of the autophagy pathway has been observed in the brains of PD patients [40].

The other half of the hemisphere was homogenized and used to measure the protein expression of some of these genes, and the inhibitory function of SR3677 was further quantified using antibody-specific immunoblotting targeting ROCK2 (Figure 1C,D). Exposure to ssEV-SR3677 decreased the expression levels of ROCK2, suggesting the inhibition of the protein by its inhibitor. Furthermore, the expression of TOM20 was significantly increased in the brains of PD mice treated with sEV-SR3677 compared to those of the saline-treated PD animals. This finding is essential, considering that mitochondrial function depends on successfully importing nuclear-encoded proteins, many of which are transported through the TOM20–TOM22 outer mitochondrial membrane import receptor machinery. Recent data suggests that post-translational modifications of α-synuclein promote its interaction with TOM20 at the outer mitochondrial membrane, inhibiting regular protein import and leading to the dysfunction and death of dopaminergic neurons [41]. Therefore, impaired mitophagy and the accumulation of damaged mitochondria play a role in the progression of the disease [42]. The expression levels of the lysosomal structural protein Lamp1 markedly decreased in brains recovered from PD animals exposed to saline and those exposed to sham sEVs or SR3677 alone compared to the results for the brains recovered from animals exposed to sEV-SR3677. This finding was the opposite of the results for the expression levels of p62/SQSTM1, a marker of the autophagic flux, which was increased in PD brains exposed to saline, while significantly decreasing by 2-fold in brains treated with sEV-SR3677, suggesting increased lysosomal fusion and the degradation of proteins. The expression of BCL2 (an inhibitor of programmed cell death and apoptosis) was also increased in PD brains exposed to sEV-SR3677 and sham sEVs compared to the results for brains recovered from PD animals exposed to saline (Figure 1C,D).

Overall, the findings showed that sEV-SR3677 administrations increased the expression of genes and proteins responsible for connecting autophagosomes and lysosomes. This suggests a general activation of the autophagy mechanism, which is implicated in the pathophysiology of PD.

### 3.2. sEVs-SR3677 Does Not Affect Inflammatory Factors in the Brain or Other Organs of the PD Mouse

**Figure 2:**
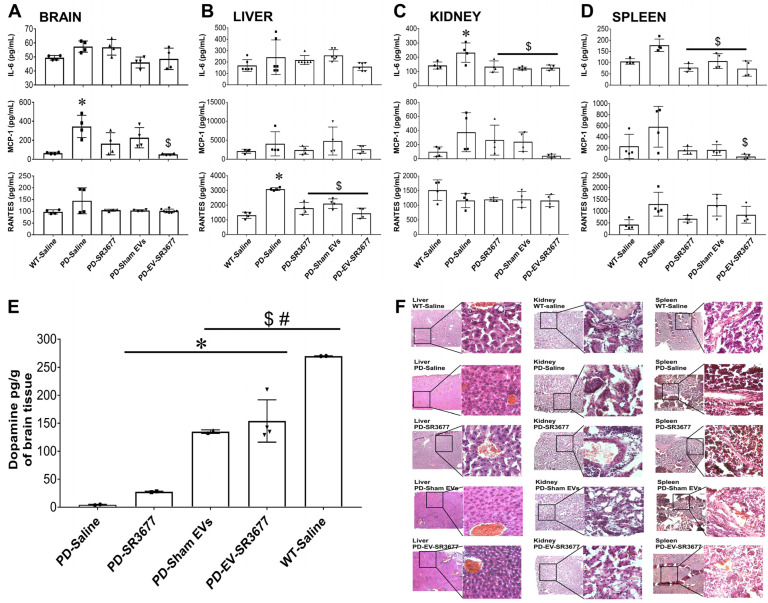
Minimal impact on inflammatory factors and elevated dopamine in brain tissues. The production of the inflammatory cytokine IL-6 and the chemokines MCP-1 and RANTES were measured in the necropsied brains (**A**), livers (**B**), kidneys (**C**), and spleens (**D**) using ELISA. The remaining brain tissues were used to measure dopamine levels by mass spectrometry (**E**), and the tissues from livers, kidneys, and spleens were used for histology and H&E staining. Representative images are shown (**F**). Images were captured using a Zeiss inverted fluorescence microscope with a 560 Axiovision camera at 5× and 40× magnification. Data (**A**–**E**) were analyzed through one- or two-way ANOVA, followed by Tukey’s multiple comparisons test, with a *p*-value of < 0.05 considered significant. * vs. WT-Saline; ^$^ vs. PD-Saline; ^#^ vs. PD-SR3677.

Since inflammation plays a key role in the pathology of PD [43], the secretion levels of inflammatory molecules in the brain and other organs were measured by ELISA (Figure 2). The production of MCP-1 increased 5-fold in the brains (Figure 2A), 2-fold in the livers (Figure 2B), 4-fold in the kidneys (Figure 2C), and 3-fold in the spleens (Figure 2D) of PD mice treated with saline, compared to the results for the healthy (wild-type) animals. The expression of RANTES in the livers (Figure 2B) and IL-6 in the spleens (Figure 2D) was significantly higher in PD mice treated with saline than in healthy (wild-type) animals. Treatment with EV-SR3677 reduced MCP-1 expression in the brains (Figure 2A), kidneys (Figure 2C), and spleens (Figure 2D) by 7-, 9-, and 12-fold, respectively, compared to the results for similar organs from PD mice treated with saline. Although the levels of inflammatory molecules in the livers and spleens of PD mice treated with saline were elevated, minimal morphological changes were observed in these tissues, as assessed by H&E (Figure 2F). Lastly, dopamine levels in the anterior brain region (including the striatum) were quantified using the liquid chromatography–tandem mass spectrometry (LC–MS/MS) method (Figure 2E). The linearity of the dopamine method was evaluated with a calibration curve constructed over the range of 5–500 pg/mL (Supplemental Material Figure S1). The dopamine concentration was low in the brains of PD animals treated with saline, while treatment with EV-SR3677 significantly (*p* < 0.001) increased dopamine levels to those of healthy (WT) animals exposed to saline (Figure 2E: last two columns).

### 3.3. sEV-SR3677 Stimulates Mitochondrial Respiration Capacity in Ex Vivo Glial Cells

**Figure 3:**
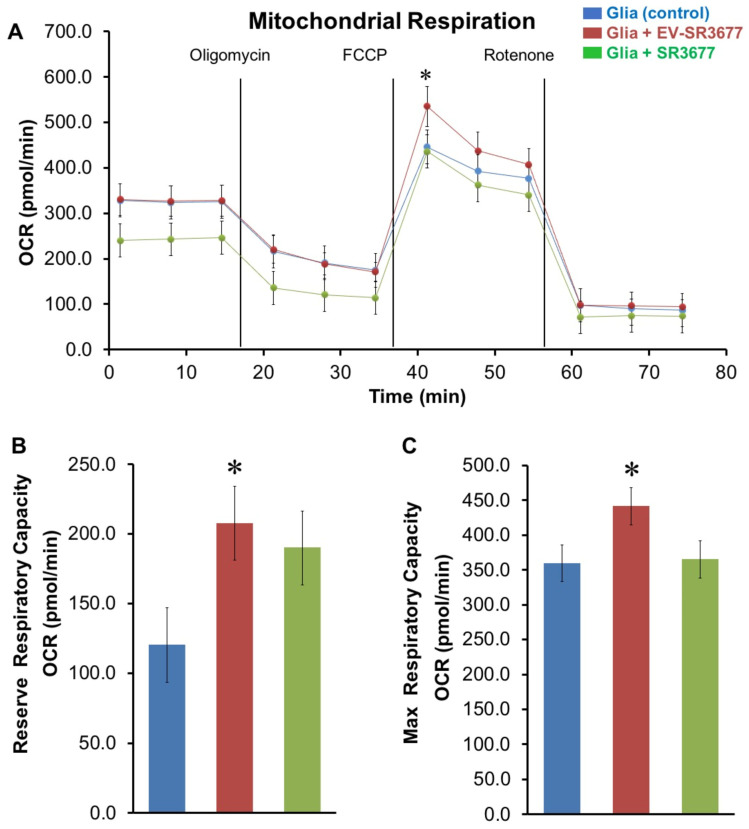
Mitochondrial stress test in mouse glia. Glial cells isolated from ex vivo mouse brain tissues were treated with consecutive injections of oligomycin (1 µM), FCCP (0.5 µM), and antimycin A (0.5 µM)/rotenone (0.5 µM). The mitochondrial respiration assay (**A**) was conducted by measuring the oxygen consumption rate (OCR), the reserve respiratory capacity (**B**), and the maximal respiratory capacity (**C**) after treatment with sEV-SR3677 or SR3677 alone. Analyses were performed using the Seahorse Wave 2.2.0 software package. A value of *p* < 0.05 was considered significant. * vs. Glia (control).

The effect of sEV-SR3677 treatment on oxygen consumption rate (OCR) and extracellular acidification rate (ECAR) values, as a direct quantification of mitochondrial respiration and glycolysis, was further measured in glial cells recovered from healthy C57BL/6J mouse pups using a Seahorse XF96 Extracellular Flux Analyzer [44]. The purity of glial cultures was determined by the percentage of GFAP (astrocytes: red) and Iba1 (microglia: green) positive cells by immunohistochemistry and was ≥80% for astrocytes and <20% for microglia (Supplemental Material Figure S2). Oxygen consumption (Figure 3A), reserve respiratory capacity (Figure 3B), maximal respiratory capacity (Figure 3C), and ECAR (Supplemental Material Figure S3) in the cells were measured after the treatment with sEV-SR3677 or SR3677 alone using the Seahorse Wave 2.2.0 software package. Treatment with sEV-SR3677 significantly enhanced reserve respiratory capacity (Figure 3B) and overall mitochondrial respiration (Figure 3C) compared to the results for the untreated glia or cells treated with SR3677 alone. Glial cells in the drug-free media were used as a control. In response to the oligomycin, ATP synthase (complex V) resulted in a reduction in mitochondrial respiration or OCR. This decrease in OCR is linked to cellular ATP production. Carbonyl cyanide-4 (trifluoromethoxy) phenyl-hydrazone (FCCP) was added as an uncoupling agent that collapses the proton gradient and disrupts the mitochondrial membrane potential. As a result, electron flow through the electron transport chain is uninhibited, and oxygen consumption by complex IV reaches the maximum. The third injection was a mixture of rotenone, a complex I inhibitor, and antimycin A, a complex III inhibitor (rotenone + antimycin A). This combination shuts down mitochondrial respiration and enables the calculation of nonmitochondrial respiration driven by processes outside the mitochondria. The findings showed a significant upregulation of oxygen consumption in cells exposed to sEV-SR3677 compared to SR3677 alone after the addition of FCCP to the cell culture, suggesting the stimulation of mitochondrial respiration (maximal oxidative phosphorylation capacity), without significantly affecting glycolysis. It is worth mentioning that in panel A, the FCCP concentration was titrated, and the lowest concentration that produced the maximal increase in OCR was selected. It is common to observe a decrease in the three subsequent measurements following FCCP injection (panel A) as the proton motive force changes with the free flow of H+ through the membrane; based on the author’s experience, this level varies according to sample or cell type. In those cells, a decrease was observed at different FCCP doses. There is a reason why maximal respiration is calculated as (maximum rate measurement after FCCP injection)—(non-mitochondrial) rather than (average rate measurement after FCCP injection)—(non-mitochondrial). Inappropriately titrated FCCP would not lead to a significant initial increase in OCR, as the reviewer mentioned. When the uncontrolled respiration threshold is reached, FCCP would completely collapse the proton motive force and consequently, shut down the electron transport chain instead of stimulating it. Before that point, the decrease in proton motive force is relatively small compared to the respiratory stimulation by FCCP. Maximal respiration varies among cell types; in this study, it increased to about 2-fold that of basal respiration, which is not considered poor; instead, increases of 2- to 3-fold are quite standard, as glial cells are not known to have very high reserves of mitochondrial respiratory capacity. It is uncommon to see stimulation exceeding five times [12,13,14,45,46,47].

## 4. Discussion

Targeting mitophagy-associated Parkinsonism using bio-inspired formulations of ROCK inhibitors provides the opportunity for treatment of this debilitating disorder. Several inhibitors of Rho-associated protein kinase are effective mitophagy promoters; among them, SR3677 demonstrated significant neuroprotective effects in vivo [48,49,50,51]. Unfortunately, delivering these small-molecule therapeutics to the brain is challenging. To address this challenge, we developed a biomimetic drug delivery system based on sEVs that incorporated SR3677 and validated the resulting formulation in mouse models of PD. SR3677 is a selective inhibitor of ROCK2. The selective inhibition of ROCK2 is particularly strong due to its high affinity for the enzyme, making it a valuable tool for studying ROCK2 function in research and in potential therapeutic applications, including for Alzheimer’s disease and the regulation of tau protein levels [52]. Specifically, SR3677 is a potent, ATP-competitive inhibitor of ROCK2 that interacts with the hydrophobic surface of the ROCK2 pocket through its benzodioxane phenyl ring. SR3677 inhibits ROCK2 by binding directly to its ATP-binding pocket, functioning as a competitive inhibitor, effectively blocking the kinase activity of ROCK2 and preventing it from phosphorylating its target proteins.

Our findings indicate that, compared to wild-type animals, PD animals treated with saline exhibited the downregulation of several genes involved in mitochondrial function and mitophagy. For instance, the aryl hydrocarbon receptor–interacting protein (AIP), a FK506-binding protein homologue, interacts with Tom20. This gene has been reported as crucial for transporting survivin to the mitochondria, facilitating its anti-apoptotic function [53]. The expressions of the *Bbc3* and *Bcl2* genes, both part of the BH3-binding domain family, were upregulated following treatment with sEVs loaded with SR3677. Members of this family can either suppress or promote cell death. Studies have demonstrated that BH3 proteins can localize to the mitochondria after receiving apoptotic signals, where they bind to Bcl-2 family members and trigger mitochondrial events related to apoptosis, including the release of cytochrome c into the cytosol [54]. Interestingly, animals treated with sham sEV showed an upregulation of the gene carnitine palmitoyltransferase 1B (*Cpt1b*). The protein encoded by this gene plays a crucial role as the rate-controlling enzyme of the long-chain fatty acid beta-oxidation pathway. Therefore, its dysregulation is linked to neurodegenerative diseases like Parkinson’s disease. Another interesting finding was the upregulation of *Opa1*, which encodes a protein called optic atrophy protein 1 (OPA1), a dynamin-related protein involved in several mitochondrial processes, including mitochondrial structure, fusion, and stability in the brains of animals treated with sEV-SR3677, demonstrating the potential therapeutic effect of this approach. The mechanochemical GTPase OPA1 modulates mitochondrial morphology as it influences the architecture of the cristae and facilitates the fusion of the mitochondrial inner membrane (IMM). Mitochondrial fusion is regulated by mitofusins (mfn) 1 and 2 at the OMM, as well as OPA1 at the inner mitochondrial membrane (IMM) [55,56,57]. The expression of *Opa1* was elevated in PD brains treated with sEV-SR3677. Consistent with this notion, it has been shown that OPA1 overexpression is beneficial in experimental models of certain diseases [58]. RhoA/ROCK signaling, essential for actin and myosin dynamics during border cell migration, may influence mitochondrial fission by regulating OPA1 expression.

*Cdkn2a* was downregulated in the brains of PD animals treated with SR3677 alone and with sEV-SR3677. The expression of the cyclin-dependent kinase inhibitor 2A (CDKN2A) gene is positively correlated with cellular senescence and has emerged as a valuable marker of both cellular senescence [59] and the pathogenesis of Parkinson’s disease, since CDKN2A is essential for regulating the cell cycle and providing protection to dopaminergic neurons in the midbrain, which are severely affected in PD. [60,61].

Compared to the results for PD animals treated with saline, the gene expression of *Dram1, Gabarap*, and *Lamp1* was upregulated in the brains of PD animals treated with sEV-SR3677 by 4-, 2-, and 3-fold, respectively. The DNA damage-regulated autophagy modulator 1 (DRAM1) is a protein that regulates autophagy and apoptosis. *Gabarap* encodes the GABA type A receptor-associated protein, which is crucial for regulating the levels of Fn14 and contributes to autophagy, as well as to the trafficking and recycling of the GABAA receptors. Studies have reported that PD symptoms develop due to the disruption of dopaminergic neurotransmitters and other neurotransmitters, such as γ-aminobutyric acid (GABA) [62,63]. Another significant finding was the upregulation of *Lamp1*, which encodes lysosomal-associated membrane proteins (LAMP). LAMP1 is a critical transmembrane glycoprotein found in lysosomes and late endosomes, playing an essential role in the function of autophagosomes and lysosomes. Rahmani, Z. et al., reported that the absence of Drosophila Lamp1, a homolog of human LAMP1 and LAMP2, significantly increases the susceptibility of flies to paraquat, a pro-oxidant associated with Parkinson’s disease. Furthermore, the loss of Lamp1 exacerbates locomotor deficits induced by the expression of the Parkinson’s-related mutant α-synuclein A30P in dopaminergic neurons, highlighting its vital role in neuroprotection [64]. Consistently, the gene expression and protein synthesis of LAMP1 were significantly downregulated in PD animals treated with saline and upregulated in PD animals treated with SR3677, sham sEV, and sEV-SR3677 (Figure 1C,D).

It has been demonstrated that in a healthy brain, damaged mitochondria are predominantly and efficiently cleared from cells via the PTEN-induced putative kinase 1 (PINK1)/Parkin-mediated mitophagy. Parkin promotes mitophagy by ubiquitinating the outer membrane proteins of the mitochondria and recruiting ubiquitin-binding autophagic components, such as histone deacetylase 6 and sequestosome-1 p62/SQSTM1 [65]. Also, PINK1 generates a novel signature on the mitochondria, thereby recruiting nuclear dot protein 52 kDa and optineurin to the mitochondria to induce mitophagy [66]. Consistent with our findings, the expression of P62 protein was decreased in brains recovered from PD animals treated with sEV-SR3677, indicative of autophagic flux [67]. PD-associated mitochondrial dysfunction presents with a variety of molecular events, including impaired mitochondrial biogenesis, increased release of free radicals, defective mitophagy and trafficking, electron transport chains dysfunction, variations in mitochondrial dynamics, calcium imbalance, neuroinflammation, and possible indirect influences on mitochondrial homeostasis from presumably unrelated pathways (e.g., α-synuclein deposition). This promotes cell death in the affected region and thereby, amplifies the inflammatory responses that further contribute to neurodegenerative diseases and neuropsychiatric disorders [68,69].

In PD, cytokines play a crucial role in the inflammatory processes that contribute to the degeneration of dopaminergic neurons in the brain. This degeneration is driven by activated microglia, which release neurotoxic factors detrimental to neuronal health [70]. Several studies have demonstrated that patients with PD show elevated levels of pro-inflammatory cytokines, including TNF-α, IL-1β, and IL-6, which are associated with disease progression and symptom severity [71]. Our findings showed a significant decrease in MCP-1 in the brains of PD animals treated with sEV-SR3677 compared to PD animals treated with saline (Figure 2A). MCP-1 serves as a significant inflammatory marker, and recent research has correlated heightened MCP-1 levels with the severity of Parkinson’s disease, as well as a possible association with non-motor symptoms, such as depression and cognitive impairment [72,73]. Although not significant, we observed a reduction in the canonical inflammatory marker IL-6 in the brains of PD animals treated with sEV-SR3677, bringing levels down to those comparable to those of wild-type mice. Several studies have confirmed that increased levels of IL-6 are associated with poorer motor function [74]. This reduction in movement abilities could be attributed to the crucial role of IL-6 in inflammation, which may have detrimental effects on muscle function and tissue repair. These findings suggest that the ROCK2 inhibitor SR3677, when used with an effective drug delivery vehicle such as small extracellular vesicles (sEVs), not only acts as a neuroprotector by promoting mitophagy but also has the potential to partially revert motor symptoms by decreasing neuroinflammation.

Current research suggests that in PD, inflammation is not limited to the substantia nigra region of the brain, where dopamine-producing neurons are found. Instead, it occurs throughout the body, suggesting a systemic inflammatory response in which the immune system is activated on a broader level, affecting areas beyond the impacted brain region [75,76]. Therefore, we evaluated the expression of pro-inflammatory cytokine and chemokines in the liver, kidney, and spleen. Consistent with previous reports, our results showed that compared to the results for wild type animals, RANTES significantly increased in the liver of PD mice treated with saline. Interestingly, in livers of PD mice treated with sEV-SR3677, RANTES significantly decreased by 2.6-fold compared to the results for PD animals treated with saline. Tang P et al. evaluated the correlation between RANTES levels and the severity of Parkinson’s disease, indicating that higher RANTES levels may reflect the severity of PD symptoms. In this context, RANTES might serve as a surrogate biomarker for assessing PD severity [77]. Conversely, in the kidneys of PD mice treated with saline, IL-6 secretion was significantly overexpressed compared to that in wild-type mice, while MCP-1 was downregulated by 9-fold in PD mice treated with sEV-SR3677 compared to the results for those treated with saline. In the context of PD, elevated IL-6 levels are strongly linked to disease progression and are believed to play a significant role in neuroinflammation. This inflammation impacts both the brain and kidneys. Several reports show that high IL-6 levels are correlated with renal dysfunction in patients with PD [78]. However, the specific mechanisms involved are still being investigated. Recent research indicates that nephropathy may be explained through mechanisms such as increased oxidative stress and damage to renal endothelial cells [79]. An additional noteworthy finding was the significantly lower levels of MCP-1 observed in the kidneys of PD mice treated with sEV-SR3677. Elevated levels of MCP-1 are strongly associated with increased inflammation and possible disease progression [80,81]. This connection arises from MCP-1’s role in attracting inflammatory cells, such as monocytes and macrophages. Monitoring MCP-1 levels in the kidney is crucial, as they may serve as a reliable biomarker for evaluating the advanced stages of PD [72,80]. This highlights the effect of sEV-SR3677 in mitigating the progression and severity of PD pathology. Although spleen analysis did not show statistical significance, the trend of elevated cytokine levels in PD mice treated with saline, compared to the reduced levels in mice treated with SR3677 alone, sham sEV, and sEV-SR3677, remained.

EVs have emerged as ideal candidates for encapsulating bioactive molecules—including proteins, peptides, siRNAs, and mRNA—to achieve precise modulation at specific cellular sites, with implications for altering genetic pathways involved in PD progression. Previous investigations by us and others have demonstrated the remarkable ability of immunocyte-derived sEVs to interact with recipient cells [82,83,84], target inflamed brain tissues via LFA1/ICAM1 interactions, and deliver their cargo [85,86,87], resulting in profound therapeutic effects in mouse models of neurodegenerative disorders [29,32,88,89]. Haney et al. previously reported [32] that EVs isolated from autologous bone marrow-derived macrophages/monocytes can reflect the properties of their parent cells and carry the same signal molecules. The proteomic landscape of EVs was characterized previously by a heterogeneous assortment of proteins, encompassing tetraspanins (e.g., CD9, CD63, CD81); endosomal-associated proteins (ALIX, TSG101); molecular chaperones such as heat-shock proteins (HSP70, HSP90); metabolic and oxidative enzymes (GAPDH, nitric oxide synthase, catalase); receptor entities (EGFR); antigen-presenting complexes such as major histocompatibility complex I–II; molecules mediating cellular adhesion, including integrins; and cytoskeletal constituents (actin, gelsolin, myosin, tubulin), alongside an array of cytosolic proteins via Western blotting [21]. Regarding drug delivery targeting by sEV nanocarriers, it is well-established that the specialized immune system cells, including monocytes, macrophages, and T cells, can accumulate in the PD brain, migrating to the sites of neuroinflammation and degeneration [90,91]. Moreover, this ability to target inflamed tissues was also shown for macrophage-derived EVs [29,32,89], which is of particular importance because the pathological process in the brain of PD patients is accompanied by chronic neuroinflammation. We also reported the lack of any systemic toxicity following EV-drug treatments in the Parkin Q311X(A) mouse model [21,32].

Here, we have successfully manufactured the sEV-SR3677 formulation and convincingly demonstrated the therapeutic efficacy of sEV-SR3677 in the PD mouse model, including enhanced expression of genes and proteins associated with mitophagy (Figure 1) and improved dopamine levels (Figure 2). Increasing the capacity of dopaminergic neurons to eliminate damaged mitochondria will lead to the development of much-needed disease-modifying therapeutics for treating PD and other diseases characterized by mitochondrial dysfunction. We used an intranasal route of drug administration because we have previously shown high accumulation levels of EV-nanocarriers in the brains of PD mice [21,32]. Our studies also showed increased oxygen consumption (Figure 3) and glycolysis in glial mitochondria (Supplemental Material Figure S3). These findings are of particular interest, considering that studies have shown abnormal glycolysis in peripheral cells in Alzheimer’s disease (AD), PD, and amyotrophic lateral sclerosis (ALS) [92]. In contrast, others showed that enhancing glycolysis attenuated PD progression in models and clinical databases [93].

In summary, our findings are significant because we developed sEV-based technology that provides crucial advantages over current delivery systems, including (i) site-specific drug delivery, administered intranasally, to target inflamed tissues, (ii) sustained drug release at those sites, (iii) improved pharmacokinetics, and (iv) the use of non-toxic biocompatible nanocarriers. Despite the importance of our pilot project, it exhibits limitations, such as the lack of clarity regarding sex differences, since only male mice were used, and the absence of measurements for compound accumulation in the brain. Another limitation was the small sample size of five animals per treatment. Furthermore, this is a preliminary study, and we did not delve deeply into the specific mechanisms by which SR3677 inhibits ROCK2 and how this inhibition enhances autophagy and mitochondrial function. Studies that include behavioral analysis and provide better insights into the role of ROCK inhibitors in the mitophagy pathway should be further investigated using animal models. Additionally, we observed that sham sEV also produced some effects that enhanced gene regulation, and the expression of proteins related to mitophagy and mitochondrial function, as well as increased dopamine production in the brain. Therefore, future studies characterizing EVs in PD are crucial for identifying novel therapeutic targets by further understanding the specific protein and nucleic acid cargo carried within these vesicles, particularly focusing on the role of ROCK2 inhibitors in restoring mitophagy and mitochondrial function.

## Data Availability

Data is contained within the article or Supplementary Material are available in section “MDPI Research Data Policies” at https://www.mdpi.com/ethics.

## Supplementary Materials

The following supporting information can be downloaded at: https://www.mdpi.com/article/10.3390/pharmaceutics17030365/s1, Figure S1. Calibration Curve Dopamine. MS/MS detection was performed via LC-TIMS-TOF MS/MS analysis. The calibration curve included six concentrations in ng/mL (ppb): CS1: 0.1 ng/mL; CS2: 0.5 ng/mL; CS3: 5 ng/mL; CS4: 20 ng/mL; CS5: 100 ng/mL; CS6: 500 ng/mL; Figure S2. The purity of glial cultures was determined by the percentage of GFAP (astrocytes: red) and Iba1 (microglia: green) positive cells by immunohistochemistry and was ≥ 80% for astrocytes and <20% for microglia. Representative image was acquired using an inverted fluorescence microscope with a 560 Axiovision camera using 40x magnification (Zeiss); Figure S3. Oligomycin (1 μg/ml final concentration), carbonyl cyanide 4-(trifluoromethoxy) phenylhydrazone (FCCP, 0.5 μM final concentration), and Rotenone plus antimycin A (0.5 μM final concentration of each) were injected sequentially. Seahorse Wave 2.2.0 software was used to analyze the data. ECAR in mpH/min was recorded; Table S1:Characterization of sEV-SR3677. Nanoparticle tracking analysis (NTA) and dynamic light scattering (DLS) revealed that a total of 7.18 × 1012 particles/mL were obtained from a single bioreactor, with an average mode size of 30.4 ± 4.1 nm and a zeta potential (Z) of −22.1 ± 0.1 mV. For drug loading, sEVs were treated with SR3677 using sonication, and then purified from unincorporated drug via size-exchange chromatography. NTA data indicated that the average size of sEV-SR3677 was 30.1 ± 5.4 nm with a concentration of 7.8 × 1012 particles/mL, achieving approximately 94% recovery. The zeta potential was minimally affected by drug loading, measuring −21.0 ± 0.7 mV. Reference [94] is cited in the Supplementary Materials.
