# Supplementary material for: Therapeutic Efficacy of Small Extracellular Vesicles Loaded with ROCK Inhibitor in Parkinson’s Disease
Source: Pharmaceutics. 2025 Mar 13;17(3):365. doi: 10.3390/pharmaceutics17030365 (PMC11944340; doi:10.3390/pharmaceutics17030365)
Supplement: Supplementary file 1 [file pharmaceutics-17-00365-s001.zip › Supplemental Material.pdf]

SUPPLEMENTAL MATERIAL

Therapeutic Efficacy of Small Extracellular Vesicles Loaded with ROCK Inhibitor in Parkinson's Disease

Candy Carbajal [1], Myosotys Rodriguez [1], Florida Owens [1], Nicole Stone [1], Dileepkumar Veeragoni [1], Rebecca Z. Fan [2], Kim Tieu [2] and Nazira El-Hage [1,*,†]

[1] Department of Cellular and Molecular Medicine, Herbert Wertheim College of Medicine, Florida International University, Miami, FL 33199, USA; ccarb060@fiu.edu (C.C.); myrodrig@fiu.edu (M.R.); fowen008@fiu.edu (F.O.); nston006@med.fiu.edu (N.S.); dveerago@fiu.edu (D.V.)

[2] Department of Environmental Health Sciences, Robert Stempel College of Public Health & Social Work, Florida International University, Miami, FL 33199, USA; zhfan@fiu.edu (R.Z.F.); ktieu@fiu.edu (K.T.)

* Correspondence: nelhage@fiu.edu; Tel.: +1-(305)-348-4246

† Current address: Department of Cellular and Molecular Medicine, Herbert Wertheim College of Medicine, Florida International University, Miami, FL 33199, USA.

MATERIALS AND METHODS

Characterization of sEV-SR3677

Nanoparticle tracking analysis (NTA) and dynamic light scattering (DLS) revealed that a total of 7.18×10^{12} particles/mL were obtained from a single bioreactor, with an average mode size of 30.4 ± 4.1 nm and a zeta potential (Z) of -22.1 ± 0.1 mV. For drug loading, sEVs were treated with SR3677 using sonication, and then purified from unincorporated drug via size-exchange chromatography. NTA data indicated that the average size of sEV-SR3677 was 30.1 ± 5.4 nm with a concentration of 7.8×10^{12} particles/mL, achieving approximately 94% recovery. The zeta potential was minimally affected by drug loading, measuring -21.0 ± 0.7 mV.

Mixed glial murine cell cultures

For primary murine glial culture, P4-P6 C57BL/6J littermates were sacrificed per IACUC guidelines. The growth medium contained DMEM (Invitrogen) supplemented with glucose (2 mg/mL; Sigma-Aldrich), Na2HCO3 (6 mM; Invitrogen), 10% heat inactivated FBS (Hyclone), and 1% penicillin/streptomycin (100 U/mL/100 μg/mL; Invitrogen). Striata were isolated and

dissociated with 0.25% trypsin and DNase (2.5 mg/ml), then centrifuged, triturated, and twice filtered through 40 μM nylon mesh. Cells were plated on poly-L-lysine (0.1mg/ml) coated plates and maintained for 5-10 days at 37°C and 5% CO2.

Immunohistochemistry

Cells were fixed in 4% paraformaldehyde, permeabilized with 0.1% Triton X-100, and blocked with 10% milk and 0.1% goat serum. Sections were immunolabeled with the Glial Fibrillary Acidic Protein (GFAP) antibody (Cat#: ab5804, Millipore, Bedford, MA, USA) at a dilution of 1:1000, and the Ionized Calcium Binding Adaptor Molecule 1 (Iba1) antibody (Cat#: sc32725, Santa Cruz Biotechnology) at a dilution of 1:100. Immunoreactivity was visualized using secondary antibodies from Molecular Probes (Carlsbad, CA, USA). 4′,6-diamidino-2-phenylindole (DAPI) was utilized to label cell nuclei. Images were analyzed using a 560 Axiovision camera (Zeiss) with an inverted fluorescence microscope.

FIGURE LEGENDS

Table S1

Sample	Mode size, nm (Mean ± SD)	ZP, mV (Mean ± SD)	Concentrations Particles/mL
sEV-SR3677	30.1 ± 5.4	-21.0 ± 0.7 mV	7.80 x 10^{12}
Sham sEVs	30.4 ± 4.1	-22.1 ± 0.1 mV	7.18 x 10^{12}

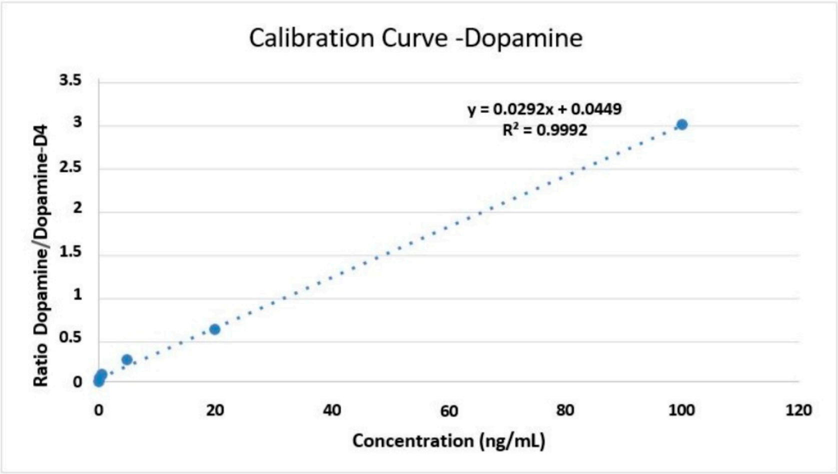

Supplemental Figure S1. Calibration Curve Dopamine. MS/MS detection was performed via LC-TIMS-TOF MS/MS analysis. The calibration curve included six concentrations in ng/mL (ppb): CS1: 0.1 ng/mL; CS2: 0.5 ng/mL; CS3: 5 ng/mL; CS4: 20 ng/mL; CS5: 100 ng/mL; CS6: 500 ng/mL.

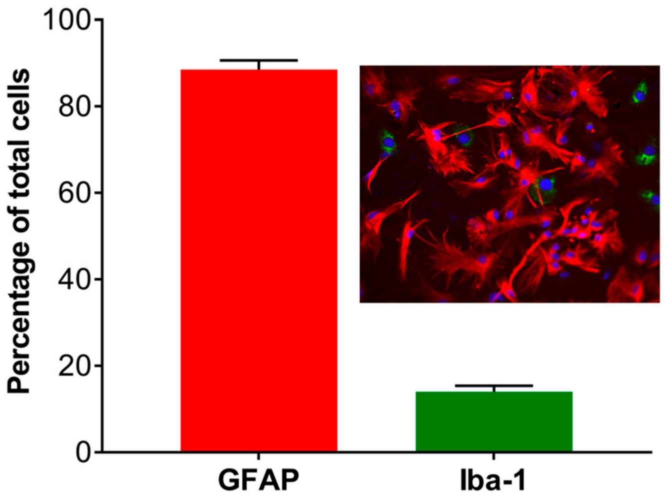

Supplemental Figure S2. The purity of glial cultures was determined by the percentage of GFAP (astrocytes: red) and Iba1 (microglia: green) positive cells by immunohistochemistry and was ≥ 80% for astrocytes and <20% for microglia. Representative image was acquired using an inverted fluorescence microscope with a 560 Axiovision camera using 40x magnification (Zeiss) [1, 2].

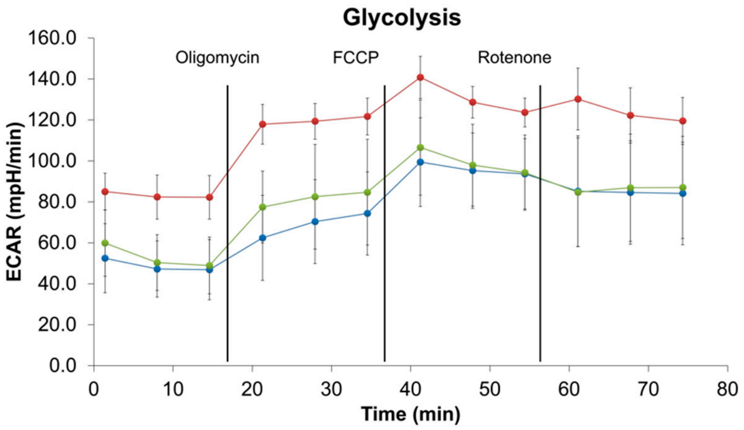

Supplemental Figure S3. Oligomycin (1 µg/ml final concentration), carbonyl cyanide 4-(trifluoromethoxy) phenylhydrazone (FCCP, 0.5 µM final concentration), and Rotenone plus antimycin A (0.5 µM final concentration of each) were injected sequentially. Seahorse Wave 2.2.0 software was used to analyze the data. ECAR in mpH/min was recorded.

References:

1. El-Hage, N., et al., *Toll-like receptor expression and activation in astroglia: differential regulation by HIV-1 Tat, gp120, and morphine.* Immunol Invest, 2011. **40**(5): p. 498-522.
2. Lapierre, J., et al., *Critical Role of Beclin1 in HIV Tat and Morphine-Induced Inflammation and Calcium Release in Glial Cells from Autophagy Deficient Mouse.* J Neuroimmune Pharmacol, 2018. **13**(3): p. 355-370.
