# Supplementary figures and images for: Therapeutic Efficacy of Small Extracellular Vesicles Loaded with ROCK Inhibitor in Parkinson’s Disease
Source: Pharmaceutics. 2025 Mar 13;17(3):365. doi: 10.3390/pharmaceutics17030365 (PMC11944340; doi:10.3390/pharmaceutics17030365)

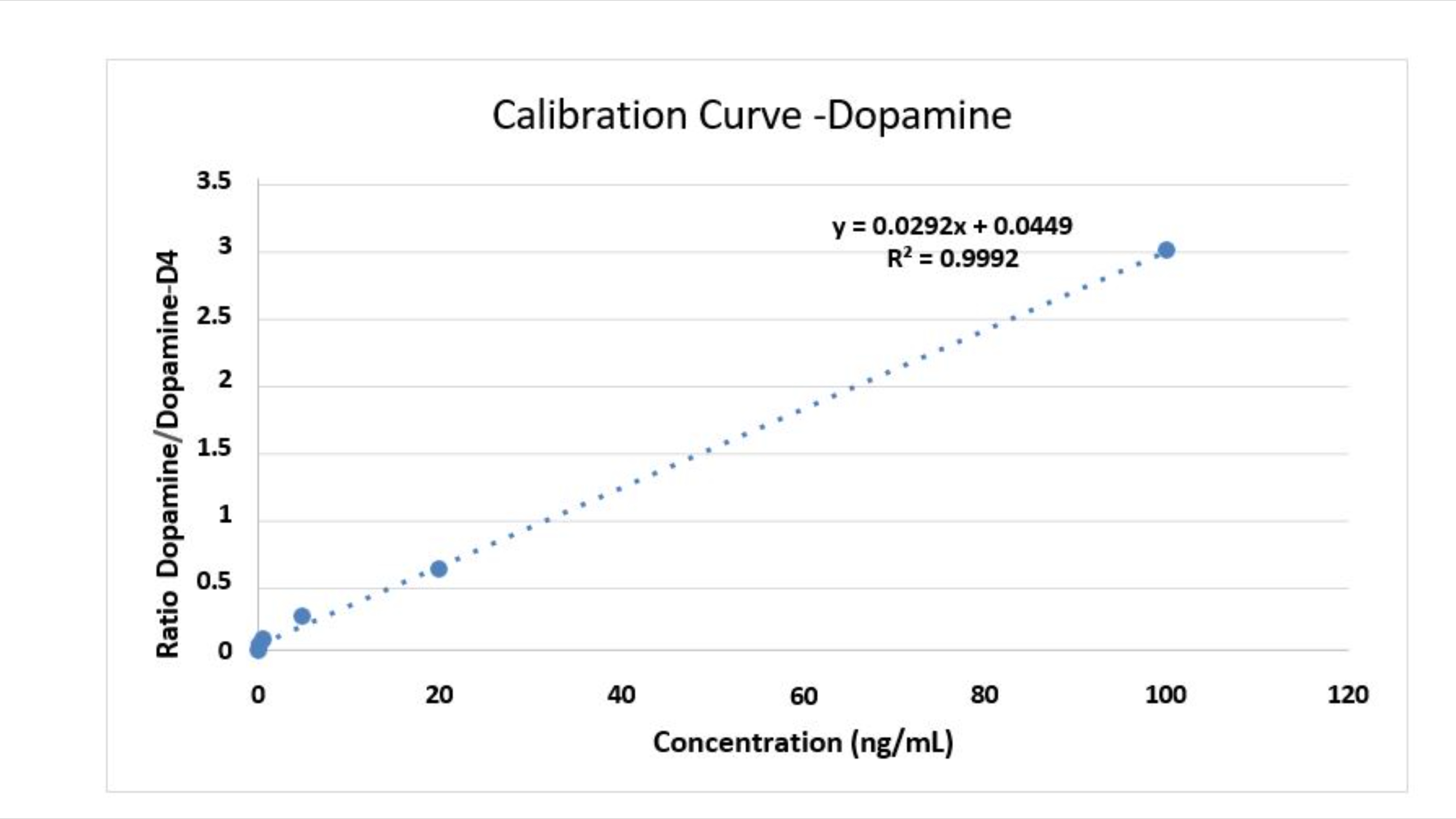

Supplement: Supplementary file 1 [file pharmaceutics-17-00365-s001.zip › Supplemental Figure S1.jpeg]

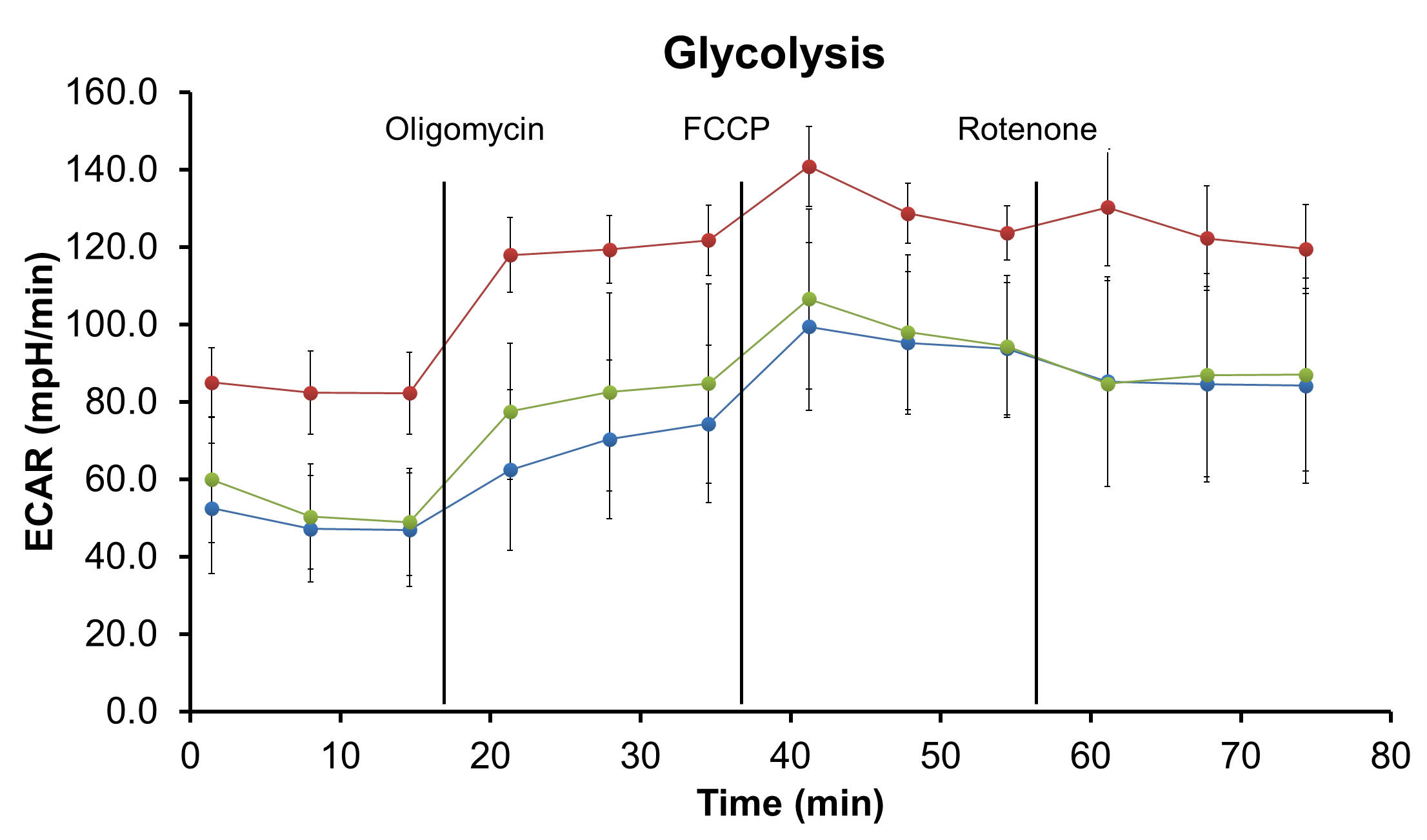

Supplement: Supplementary file 1 [file pharmaceutics-17-00365-s001.zip › Supplemental Figure S3.tif]

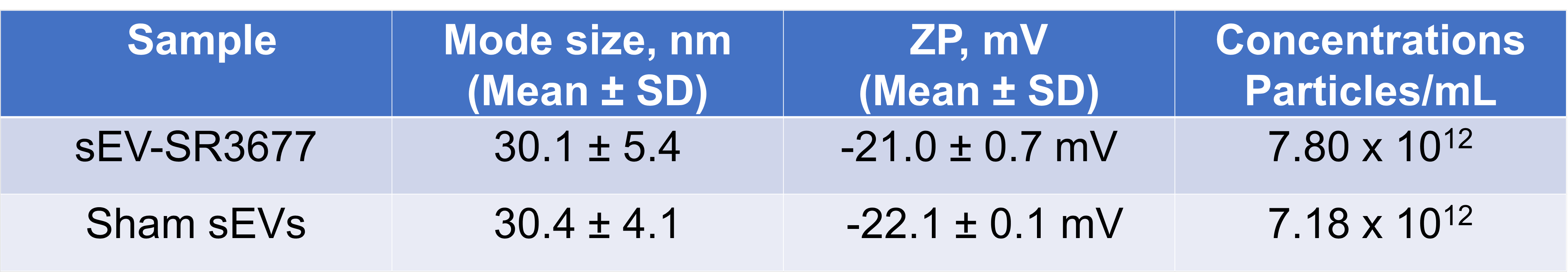

Supplement: Supplementary file 1 [file pharmaceutics-17-00365-s001.zip › Table S1.tiff]
